# High Level of Serum and Cerebrospinal Fluid of Heparan Sulfate and Hyaluronic Acid Might Be a Biomarker of Severity of Neuromyelitis Optica

**DOI:** 10.3389/fimmu.2021.705536

**Published:** 2021-07-22

**Authors:** Qi Zhang, Shanshan Pei, Zheyi Zhou, Zhanhang Wang, Yu Peng, Jinyu Chen, Honghao Wang

**Affiliations:** ^1^ Department of Neurology, The First Affiliated Hospital of Zhengzhou University, Zhengzhou, China; ^2^ Department of Neurology, Nanfang Hospital, Southern Medical University, Guangzhou, China; ^3^ Department of Neurology, Liuzhou Traditional Chinese Medical Hospital, The Third Affiliated Hospital of Guangxi University of Chinese, Liuzhou, China; ^4^ Department of Neurology, Guangdong 999 Brain Hospital, Guangzhou, China

**Keywords:** glycocalyx, blood-brain barrier, heparan sulfate, hyaluronic acid, neuromyelitis optica

## Abstract

**Background:**

Neuromyelitis optica (NMO), multiple sclerosis (MS) and autoimmune glial fibrillary acidic protein (GFAP) astrocytopathy are idiopathic inflammatory demyelinating diseases (IIDDs) that mainly present as encephalomyelitis. Heparan sulfate (HS) and hyaluronic acid (HA) are two components of glycocalyx, a carbohydrate-rich layer on the surface of blood vessels that mediates interaction with blood. Degradation of glycocalyx in NMO is poorly understood.

**Purpose:**

To detect the serum and cerebrospinal fluid (CSF) levels of shed HS and HA and to correlate these levels with disease severity to determine their diagnostic value.

**Methods:**

We obtained serum and CSF samples from 24 NMO patients, 15 MS patients, 10 autoimmune GFAP astrocytopathy patients, and 18 controls without non-inflammatory neurological diseases. Soluble HS and HA, and IFNγ, IL17A, and matrix metalloproteinase (MMP) 1 were detected *via* ELISA.

**Results:**

Serum and CSF levels of HS, HA and related cytokines but not of plasma MMP1 were significantly elevated in these diseases. Notably, HS and HA levels were positively correlated with Expanded Disability Status Scale scores.

**Conclusions:**

Our results indicate glycocalyx degradation and inflammation in NMO, MS and autoimmune GFAP astrocytopathy. Moreover, increased shedding of HS or HA may indicate a worse clinical situation. Furthermore, therapeutic strategies that protect glycocalyx may be effective in these diseases.

## Introduction

Idiopathic inflammatory demyelinating diseases (IIDDs) are devastating neurological diseases that affect both neurological and psychiatric functions and lead to poor quality of life (1). Neuromyelitis optica (NMO) and multiple sclerosis (MS) are two representative immune-mediated IIDDs (2). Recently, a new IIDD with unclear pathogenesis and no accurate diagnostic criteria was identified and defined as autoimmune GFAP astrocytopathy (3). Typically, the pathological changes of these diseases involve lymphocyte infiltration, astrocytopathy, and autoantibodies targeting receptors in the brain or spinal cord (4–6).

The blood-brain barrier (BBB) has long been accepted to prevent entry of immune cells and antibodies into the CNS (7). In IIDDs, neuro-inflammation occurs causing breakdown of the BBB (8) and the interplay between neuro-inflammation and BBB dysregulation can result in serious neurological disturbance (9–11). The condition of the BBB in NMO, MS and autoimmune GFAP astrocytopathy is not well described, and investigation of this issue may facilitate understanding of these diseases and indicate novel therapies. Glycocalyx, a delicate membrane-bound network attached to the luminal side of the BBB endothelium, is the first barrier between blood and the BBB (12). Thus, levels of glycocalyx shedding can quickly reflect the extent of BBB damage.

HS and HA are two representative glycosaminoglycans present in glycocalyx that can indicate endothelial glycocalyx degradation (12–14). Under pathological conditions, such as inflammation, hypertension or edema, glycocalyx components can be rapidly fragmented and released into the CSF or bloodstream (15–17). Indeed, previous studies of non-IIDDs, such as sepsis, brain edema, stroke, and trauma, have confirmed the shedding of HS and HA in the acute stage (18–20). In response to pathological conditions, extracellular proteases are rapidly activated and mediate the shedding of glycocalyx components, resulting in vascular permeability barrier breakdown, mechanotransduction impairment and endothelial cell dysfunction. These changes further enhance glycocalyx degradation in a feed-forward manner (20). To date, most studies that have assayed glycocalyx have focused on vascular diseases and cancer. Some studies, however, have linked glycocalyx components with encephalitis in NMO, experimental autoimmune *encephalomyelitis*, and anti-NMDA receptor encephalitis (21–23), although a holistic understanding of glycocalyx degradation in IIDDs is lacking.

In the early stage of an immune response, T cells secrete pro-inflammatory cytokines that stimulate the generation and maturation of matrix metalloproteinases (MMPs), which can act on glycocalyx (24–26). Thus, plasma and CSF concentrations of HS and HA can reflect glycocalyx damage and, indirectly, disruption of the BBB in the acute stage of a disease. No studies have detailed the shedding of glycosaminoglycans, particularly HS and HA, into CSF or serum in patients with NMO.

Herein, we focus on the role of glycocalyx in NMO, MS and autoimmune GFAP astrocytopathy. To assess the severity of BBB injury on the severity of these neurological disorders, and to identify potential factors affecting the integrity of BBB glycocalyx, we detected the levels of two glycocalyx molecules, HS and HA, in both CSF and serum. We then correlated these levels with CSF levels of pro-inflammatory factors IFNγ and IL17A, the sheddase, MMP1, and Expanded Disability Status Scale (EDSS)·scores. CSF and plasma concentrations of HS and HA, and levels of CSF IFNγ, IL17A, and MMP1 were significantly elevated in these three idiopathic inflammatory demyelinating diseases. More importantly, the levels of shed glycocalyx molecules in CSF positively affected the severity of NMO, MS and autoimmune GFAP astrocytopathy, and the degree of inflammation may aggravate the disruption of the blood-brain barrier. However, the dynamic concentrations of HS and HA and their relationships with the severity of these disorders remain unknown. Of note, CSF HS and HA may be reliable markers for the diagnosis of NMO. Furthermore, therapeutic strategies focused on preservation of glycocalyx may improve outcomes of these neurological disorders.

## Materials and Methods

### Patients and Clinical Assessments

All subjects were enrolled from the Department of Neurology of Nanfang Hospital, Southern Medical University, China, including 24 NMO patients, 15 MS patients, 10 patients with autoimmune GFAP astrocytopathy and 18 controls without inflammatory or autoimmune neurological diseases (peripheral neuropathy=10, movement disorder=6, Alzheimer’s disease=2). Diagnoses of autoimmune encephalitis were confirmed by two doctors on the basis of diagnostic criteria (3, 27, 28). All controls were negative for specific CSF and serum antibodies. EDSS scores were used to evaluate disease severity. Relevant demographic and medical data were also collected and are shown in **Table 1**. This study was approved by the Ethics Committee of the Nan Fang Hospital and all subjects provided informed consent.

**Table 1:** Demographic and clinical features of the patients and controls.

	CLTs (n = 18)	NMO (n = 24)	MS (n=15)	autoimmune GFAP astrocytopathy (n = 10)
Gender (female/male)	11/7	16/8	11/4	6/4
Age (years)^a^	35.43±10.32	34.37±15.24	36.27±14.75	33.46±17.27
EDSS scores^b^	–	4.0 (3.13, 4.88)	2.5 (2.00, 3.00)	2.75 (2.38, 3.50)
Serum HS (ng/mL)^b^	236.8 (164.2, 320.5)	429.4 (292.7, 523.8)***	321.6 (254.6, 390.0)*	395.8 (316.3,508.4)***
CSF HS (ng/mL)^b^	96.9 (74.9, 135.2)	219.4 (148.2, 272.5)***	183.7 (133.5, 202.4)***	207.6 (158.2,259.7)***
Serum HA (ng/mL)^b^	26.5 (24.4, 37.2)	70.4 (50.0,88.4)***	66.8 (51.10, 85.9)***	71.9 (48.6, 76.9)***
CSF HA (ng/mL)^b^	32.5 (26.9, 36.0)	48.1 (40.75, 57.02)***	49.04 (39.71, 56.14)***	47.5 (28.6, 61.6)
Serum IFN-γ (pg/mL)^b^	8.2 (5.7, 14.2)	63.6 (37.6, 93.0)***	54.4 (34.9, 94.8)***	57.2 (41.6, 84.0)***
CSF IFN-γ (pg/mL)^b^	8.7 (5.4,12.6)	73.0 (43.5, 86.4)***	44.8 (24.5, 80.6)***	38.8 (29.4, 58.6)***
Serum IL-17A (pg/mL)^b^	2.7 (2.55,3.2)	5.5 (4.5, 7.7)***	5.3 (3.7,9.2)***	7.0 (4.5, 7.6)***
CSF IL-17A (pg/mL)^b^	3.2 (3.1, 3.4)	4.21(3.4,10.2)***	5.2 (4.6,8.6)***	6.1 (3.7, 10.3)***
Serum MMP-1 (pg/mL)^b^	598.8 (236.3,1050.0)	548.3 (341.5,1512.0)	992.7 (491.2,2837.0)	631.1 (354.3, 976.9)
CSF MMP-1 (pg/mL)^b^	14.8 (13.3,15.7)	17.8 (15.3, 23.7)**	19.28 (16.49, 20.4)***	22.6 (18.3, 30.8)***
NMO-IgG				
Positive	0	23	0	0
GFAP-IgG				
Positive	0	0	0	10

Age (years) refers to age at sample collection. CTLs, controls; n, number; IL, interleukin; TNF, tumor necrosis factor; CSF, Cerebrospinal Fluid; EDSS, Expanded Disability Status Scale; NA, not available; HS, heparan sulphate; HA, hyaluronic acid; NMO, neuromyelitis optica; MS, multiple sclerosis. GFAP, glial fibrillary acidic protein.

^a^Data were presented as mean ± SD. SD, standard deviations.

^b^Data were presented as medians (IQRs-interquartile ranges).

P values were calculated from the difference between CLTs and NMO, MS or autoimmune GFAP astrocytopathy.*P < 0.05, **P < 0.01, ***P < 0.001.

### Measurement of HS, HA and Related Cytokines

We obtained CSF and serum samples from all subjects within 3 days of admission and before immunotherapy therapies were commenced. All samples were centrifuged at 1,000 g for 10 min. Then the supernatant was packed into polypropylene tubes and stored at -80°C until the detection. Take one small tube for each test to avoid repeated freezing and thawing. Enzyme-Linked Immunosorbent Assay (ELISA) kits were used to measure the concentrations of HS (ELH-CD44-1, RayBiotech, Atlanta, USA), HA (DHYAL0, R&D, Minnesota, USA), IFNγ, IL17A and MMP1 (IFNγ: KSC4021, IL17A: BMS2017, MMP1: EHMMP1, ThermoFisher, Massachusetts, USA). All detections were performed in accordance with the manufacturer’s instructions and every standard and sample were assayed in duplicate.

### Statistical Analyses

All statistical analyses were conducted using SPSS version 24.0 (IBM, Armonk, NY, US). Data are displayed as the mean ± SD or the median with interquartile range according to normality test results. The Kruskal–Wallis test was used to analyze the differences in CSF HS, CSF HA, SE HS and SE HA levels among the subgroups. The Pearson’s test were used to evaluate the correlations between CSF HS, CSF HA, SE HS and SE HA levels and EDSS scores. The Spearman test was used to evaluate correlations between CSF glycocalyx molecules and other cytokine parameters. p<0.05 was taken as statistically significant. Graphs were plotted using GraphPad Prism 9 (GraphPad, La Jolla, CA, US). All analyses were performed in a blinded manner.

## Results

### Demographic and Clinical Characteristics

The data from patients with NMO (n = 24), MS (n = 15), autoimmune GFAP astrocytopathy (n=10) and 18 controls are presented in **Table 1**. The median EDSS score (with IQR) was 4.0 (3.13, 4.88) for the NMO group, 2.5 (2.00, 3.00) for the MS group and 2.75 (2.38, 3.50) for the autoimmune GFAP astrocytopathy group (**Table 1**). Among the patients with autoimmune GFAP astrocytopathy, no patients with tumors were identified after routine tumor screening. There were no statistically significant differences in sex or age among groups.

### Increased HS, HA and Related Cytokine Levels in Serum and CSF in Patients With NMO, MS and Autoimmune GFAP Astrocytopathy

**Figure 1:**
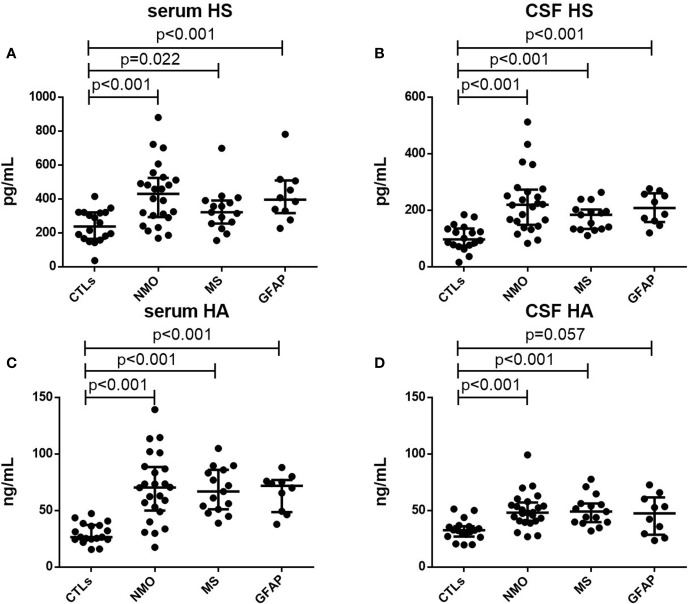
Serum and CSF levels of HS and HA. Serum levels of HS **(A)** and HA **(C)** in controls (CTLs) and patients with NMO, MS and autoimmune GFAP astrocytopathy. CFS levels of HS **(B)** and HA **(D)** in controls (CTLs) and patients with NMO, MS and autoimmune GFAP astrocytopathy. p-values are indicated within each analysis.

As shown in **Table 1**, the median concentrations of IFNγ and IL17A in CSF and serum of NMO, MS and autoimmune GFAP astrocytopathy patients were higher than those of the control group (All p<0.001). For MMP1, CSF concentrations but not serum were elevated in patients with NMO, MS and autoimmune GFAP astrocytopathy compared with those of the control group(CSF: p<0.01; Serum: p>0.05). To further assess the severity of BBB injury in these autoimmune encephalitis patients, we compared the levels of HS and HA among patients with NMO, MS and autoimmune GFAP astrocytopathy and controls. Concentrations of HS and HA in plasma and CSF were elevated in the autoimmune encephalitis group compared with those in the control group (serum HS: NMO p<0.001, MS p=0.022, GFAP p<0.001, respectively, **Figure 1A**; CSF HS: NMO p<0.001, MS p<0.001, GFAP p< 0.001, respectively, **Figure 1B**. serum HA: NMO p < 0.001, MS p<0.001, GFAP p<0.001, respectively, **Figure 1C**; CSF HA: NMO p<0.001, MS p<0.001, GFAP p=0.057, respectively, **Figure 1D**). However, no significant differences in levels of serum or CSF glycocalyx molecules or other cytokine parameters were found among NMO, MS and autoimmune GFAP astrocytopathy patients. To assess relationships between the levels of glycocalyx molecules in CSF and serum, correlation tests were performed in autoimmune encephalitis subgroups. Positive correlation between CSF and serum levels was only present for HS in NMO and HA in MS (NMO HS: p=0.029, r=0.447; MS HA: p=0.005, r=0.696).

### Correlations Between CSF and Plasma HS and HA Levels and EDSS Scores

**Figure 2:**
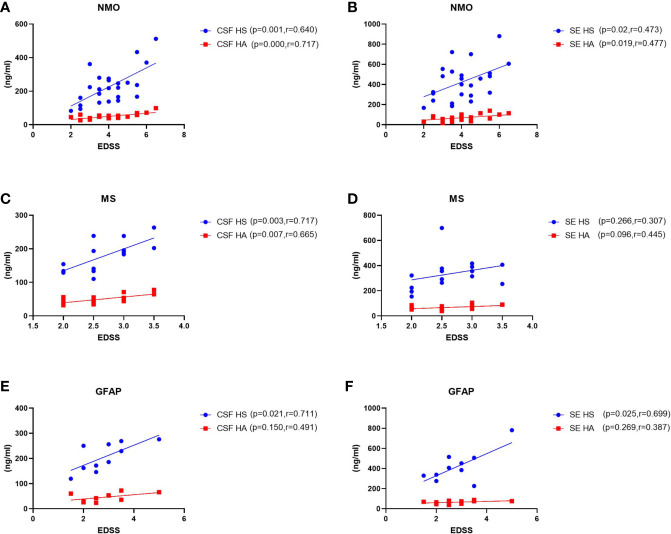
Correlations between levels of CSF and plasma HS and HA and EDSS scores in patients with NMO, MS and GFAP encephalitis. **(A–B)** In NMO, the CSF and plasma levels of HS and HA were positively correlated with EDSS scores. **(C–D)** In MS, the levels of HS and HA in CSF were significantly correlated with EDSS scores, while no correlations were found between plasma HS or HA levels and EDSS scores. **(E–F)** In autoimmune GFAP astrocytopathy, the CSF and plasma levels of HS were significantly correlated with EDSS scores, while no correlations were found between CSF and plasma HA levels and EDSS scores. The p and r-values are indicated within each analysis.

To assess possible links between CSF and plasma glycocalyx levels and the severity of these three disorders, we examined correlations among them (**Figure 2**). In patients with NMO, MS or autoimmune GFAP astrocytopathy, we detected a significant positive correlation between concentrations of CSF HS and HA and EDSS scores (NMO HS: p=0.001, r=0.640; MS HS: p=0.003, r=0.717; autoimmune GFAP astrocytopathy HS: p=0.021, r=0.711; NMO HA: p=0.000, r=0.717; MS HA: p=0.007, r=665). For serum levels and EDSS scores, we only found significant correlation between serum HS and HA and EDSS scores in the NMO and GFAP group (NMO SE HS: p=0.02, r=0.473; NMO SE HA: p=0.019, r=0.477; GFAP SE HS: p=0.025, r=0.699). Positive correlations between other serum levels and EDSS scores were detected but they did not reach statistical significance (MS SE HA: p=0.096, r=0.445).

### Associations Between CSF Glycocalyx Molecules and Other Cytokine Parameters

**Figure 3:**
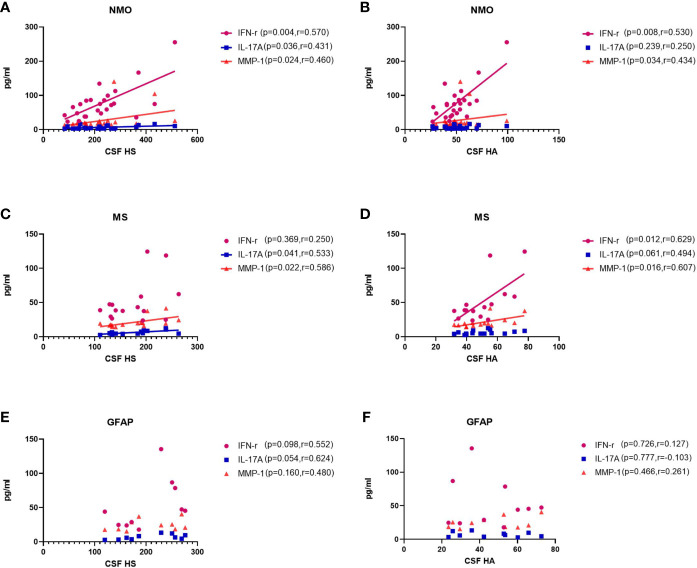
Correlations between the CSF levels of HA and HS and CSF levels of IFNγ, IL17, and MMP1 in patients with NMO, MS and autoimmune GFAP astrocytopathy. **(A, B)** In NMO, the CSF levels of HS were positively correlated with IFNγ and MMP1, while the CSF levels of HA was positively correlated with MMP1. **(C, D)** In MS, the CSF levels of HS were positively correlated with MMP1, while the CSF levels of HA was positively correlated with IFNγ. **(E, F)** In autoimmune GFAP astrocytopathy, the CSF levels of HS were positively correlated with MMP1, while the CSF levels of HA was positively correlated with IL17. The *p* and *r-*values are indicated within each analysis.

Previous studies have found elevated levels of pro-inflammatory cytokines in CSF of patients with NMO, MS and autoimmune GFAP astrocytopathy. Here, we detected associations among CSF glycocalyx molecules and IFNγ, IL17A and MMP1 **(** **Figure 3**). Interestingly, there was a general positive correlation between HS and IFNγ, IL17A, and MMP1 concentrations in the CSF of NMO patients (IFNγ, p=0.004, r=0.570; IL17A, p=0.036, r=0.431; MMP1, p=0.024, r=0.460.) (**Figure 3A**). Moreover, significant positive correlations were also found between CSF HA and IFNγ (p=0.008, r=0.530) and CSF HA and MMP1 (p=0.034, r=0.434) in the NMO group (**Figure 3B**). CSF HS and IL17A (p=0.041, r=0.533), CSF HS and MMP1 (p=0.022, r=0.586),CSF HA and IFNγ (p=0.012, r=0.629), CSF HA and MMP1 (p=0.016, r=0.607) were also found significant positive correlations in the MS group (**Figures 3C, D**). In the GFAP group, there was no significant correlation between CSF glycocalyx molecules and other cytokine parameters (**Figures 3E, F**).

### Receiver Operating Characteristic (ROC) Curve Analysis

**Figure 4:**
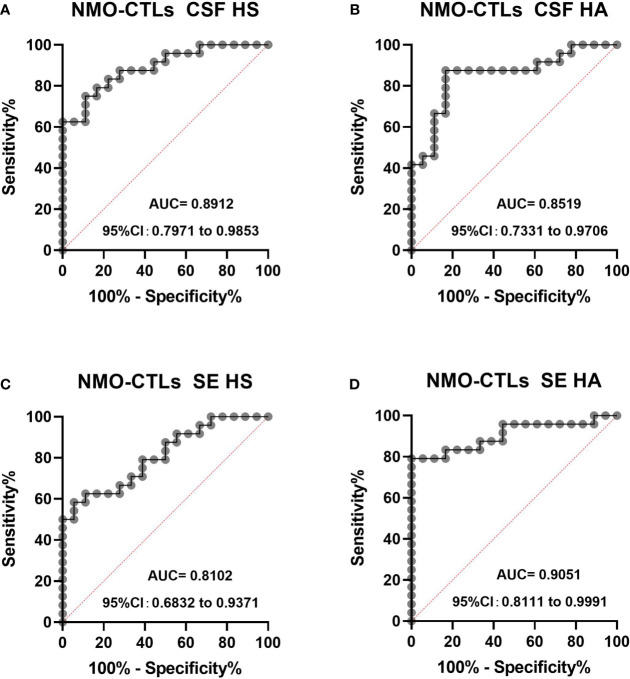
The ROC curve analysis of CSF and plasma HS and HA in NMO patients to distinguish from CTL **(A–D)**. The area under curve (AUC) for CSF HS in NMO patients to distinguish from CTL patients was 0.819 (95% CI: 0.791-0.9853, P <0.0001) **(A)**, the AUC for CSF HA was 0.8519 (95% CI: 0.7331-0.9707, P= 0.0001) **(B)**. The AUC for SE HS was 0.8102 (95% CI: 0.6832-0.9371) **(C)**, the AUC for SE HA was 0.905 (95% CI: 0.811-0.9991, P <0.0001) **(D)**. CI, confidence interval.

We performed ROC analysis for CSF and plasma HS and HA to distinguish NMO patients from control patients in **Figure 4**. The area under curve (AUC) for CSF HS in NMO patients to distinguish from CTL patients was 0.819 (95% CI: 0.791-0.9853, P <0.0001) (**Figure 4A**), the AUC for CSF HA was 0.8519 (95% CI: 0.7331-0.9707, P= 0.0001) (**Figure 4B**). The AUC for SE HS was 0.8102 (95% CI: 0.6832-0.9371) (**Figure 4C**), the AUC for SE HA was 0.905((95% CI: 0.811-0.9991, P <0.0001) (**Figure 4D**).

## Discussion

This study focused on the extent of GLX disruption in three typical idiopathic inflammatory demyelinating diseases, NMO, MS and autoimmune GFAP astrocytopathy. We determined the levels of HS and HA shed into in plasma and CSF and we propose diagnostic roles for HS and HA in these three autoimmune diseases.

The BBB prevents certain plasma components, such as inflammatory factors and immune cells, from entering the central nervous system (15, 17, 29) and, therefore, plays a crucial role in maintaining cerebral homeostasis. Evidence indicates breakdown of the BBB in autoimmune diseases of the CNS (30), but the early changes to the BBB milieu are poorly characterized in idiopathic inflammatory demyelinating diseases (IIDDs), especially in the newly identified autoimmune GFAP astrocytopathy. Importantly, effective therapies that protect the BBB in other diseases, such as brain edema, indicate potential therapies for NMO, MS and autoimmune GFAP astrocytopathy (20).

Glycocalyx, a jelly-like layer, covers the luminal surface of the endothelium and is thought to be the first defensive layer of the BBB (12, 20, 31). If this surface matrix is disrupted, the homeostasis of endothelial permeability is damaged, resulting in leakage of inflammatory cytokines, immune cells and even antibodies from serum into cerebrospinal fluid, which aggravates inflammation and the severity of diseases (11, 32, 33). Thus, high concentrations of soluble glycocalyx components in body fluids, especially CSF, indicate glycocalyx damage and, indirectly, disruption of the BBB. Here, we show substantial increases in soluble HS and HA in plasma and CSF in the acute stages of NMO, MS and autoimmune GFAP astrocytopathy, indicating degradation and disruption of glycocalyx and the BBB. Notably, the CSF levels of both HS and HA were positively associated with the severity of the diseases. Moreover, under inflammatory conditions, glycocalyx shedding occurs in response to mediators and activation of MMPs (24, 26, 34, 35), resulting in release of HS and HA fragments into the circulation. We also found elevated levels of MMP1 in CSF and CSF HS and HA were positively associated with NMO, MS and autoimmune GFAP astrocytopathy.

The current study also focused on the extent of neuro-inflammation. The release of inflammation-mediated cytokines can stimulate the process of glycocalyx shedding (15, 17). Of note, IL17A and IFNγ, two pro-inflammatory factors involved in many autoimmune disorders, were activated in NMO, MS and autoimmune GFAP astrocytopathy (36). Moreover, MMPs can be induced under inflammatory conditions (37, 38). Notably, positive correlations were found between concentrations of CSF HS and those of IFNγ, IL17A, and MMP1 in these diseases. Therefore, the release of pro-inflammatory cytokines may interact with MMPs and result in glycocalyx dysfunction. Conversely, disruption of endothelial glycocalyx can cause damage to the BBB, which in turn exacerbates the extent of inflammation and the activation of MMPs.

Difficulties with patient follow up meant that we were unable to perform certain detailed assessments, such as dynamic monitoring of HS and HA shedding, and determining the association between the degree of glycocalyx destruction and disease severity in different courses of the diseases. Therefore, further clinical and animal studies are warranted to address these issues.

In conclusion, we have demonstrated elevated levels of shed HS and HA in plasma and CSF, indicating that glycocalyx degradation occurs in the acute stage of NMO. Moreover, CSF HS and HA levels were correlated with the severity of these diseases. Importantly, CSF HS and HA are potential indicators for the diagnosis of these three idiopathic inflammatory demyelinating diseases. We also showed activation of inflammation. Further investigations measuring dynamic glycocalyx shedding in different courses of diseases are worthwhile. Importantly, therapeutic strategies that protect glycocalyx may be effective for these diseases.

## Data Availability Statement

The datasets presented in this study can be found in online repositories. The names of the repository/repositories and accession number(s) can be found in the article/supplementary material.

## Ethics Statement

This study was approved by the Ethics Committee of the Nan Fang Hospital and all the above subjects have signed the informed consent. Written informed consent to participate in this study was provided by the participants’ legal guardian/next of kin.

## Author Contributions

HW conceived this study and designed the experiments. QZ, SP, JC, YP, ZZ and ZW collected the samples and clinical data. QZ, JC, SP performed the experiments, analyzed the data, and wrote the manuscript. All authors contributed to the article and approved the submitted version.

## Funding

Financial support came from the National Natural Science Foundation of China (No.81301028, 8167395, 82060905, 81760902, 81901239), Guangxi Natural Fund Project (No. 2019GXNSFAA245079), Guangdong Provincial Science and Technology plan projects (2017A020215182), Natural Science Foundation of Guangdong Province (2019A1515011434), and Guangzhou Science and Technology Plan Project (202102020434).

## Conflict of Interest

The authors declare that the research was conducted in the absence of any commercial or financial relationships that could be construed as a potential conflict of interest.

## Abbreviations

CNS, central nervous system; CSF, cerebrospinal fluid; EDSS, Expanded Disability Status Scale; HA, Hyaluronic acid; HS, Heparan sulfate; MS, multiple sclerosis; NMO, Neuromyelitis optica; GFAP, autoimmune glial fibrillary acidic protein.
